# Genetic features of BEL-1-producing and KPC-2-producing *E. coli* from hospital wastewater: human source or sewages adaptation

**DOI:** 10.1007/s11356-024-33875-w

**Published:** 2024-06-24

**Authors:** Laura Romero-Oraá, Marina R. Pulido, Fatima Galán, María Victoria García Palacios, Alvaro Pascual, Lorena López-Cerero

**Affiliations:** 1 Unidad Clínica de Enfermedades Infecciosas y Microbiología, Hospital Universitario Virgen Macarena, Instituto de Biomedicina de Sevilla IBIS, Seville, Spain; 2 Departamento de Microbiología, Universidad de Sevilla, Avda Dr. Fedriani S/N. 41009, Seville, Spain; 3 Unidad de Microbiología, Hospital Universitario Puerta del Mar, Cádiz, Spain; 4 Servicio de Medicina Preventiva, Hospital Universitario Puerta del Mar, Cádiz, Spain; 5 CIBER de Enfermedades Infecciosas, Instituto de Salud Carlos III, Madrid, Spain

**Keywords:** KPC-2, BEL-1, Hospital sewages, P1 bacteriophage

## Abstract

Hospital sewage is an ecosystem that facilitates the transfer of antibiotic and heavy metal resistance genes and the interaction of human and environmental bacteria. In this environment, we have detected the presence of 7 KPC-2 and BEL-1 co-producing *E. coli* isolates of two different clones over a 10-month period in the same hospital. All isolates carried *bla*_KPC-2_ and the operon *mer* on the same *Inc*P plasmid of similar size and an *Inc*N plasmid of different size each clone carrying *bla*_BEL-1_. Both IncN-blaBEL-1 plasmids shared a 77 kb region containing *bla*_BEL-1_ alongside with *fosE*, *bla*_OXA-10_ and *aac(6’)-1b* genes in a class 3 integron within a Tn*3* transposon. The major *Inc*N plasmid contained in addition a region homolog to P1-like bacteriophage RCS47, including the lytic RepL and lysogenic proteins, but other phage regions were incomplete. The characters such as the temporal persistence in sewage, the absence of colonized patients in the hospital or in the region, the presence of a p1 phage-plasmid fusion and the infrequent class 3 integron as genetic platform would indicate that BEL-1-producing isolates could have been generated in situ by adaptation to human sewage. Part of the microbiota in these discharges could be explained by the interactions of sewage ecosystems and not derive directly from the hospital.

**Supplementary Information:**

The online version contains supplementary material available at 10.1007/s11356-024-33875-w.

## Introduction

Hospital effluents could play a role in the generation and spread of antibiotic-resistant determinants (Korzeniewska and Harnisz 2013). In these effluents, the presence of antibiotic-resistant bacteria from patients is combined with the selective pressure of spilled antibiotics and heavy metals (Hubeny et al. 2021). Many resistant genes are located on mobile genetic elements such as plasmids, transposons, and integrons (Brolund and Sandegren 2016). Generally, spontaneous frequency of plasmid conjugation is low; however, various compounds present in hospital effluents such as disinfectants, heavy metals, human treatments such as anticonvulsants, antiepileptic, and antibiotics, have been reported to promote plasmid conjugation rate (Liu et al. 2022). The concentrations of antibiotics and heavy metals required to maintain plasmid-carrying bacteria are subinhibitory in most cases (Gullberg et al. 2014). In this ecosystem, bacteria of clinical origin adapted to this aquatic environment coexist with environmental bacteria and other types of microorganisms in biofilms that favor genetic exchange and selection with subinhibitory substances. Due to the favorable conditions for genetic exchange, new combinations of species and resistance determinants of clinical or non-clinical origin, as well as mobile elements, can be found in hospital sewages.

BEL-1 (Belgium extended beta-lactamase) is a clavulanic acid-inhibited expanded-spectrum ß-lactamase (ESBL) detected in a *Pseudomonas aeruginosa* strain isolated from a scrotal swab of a 72-year-old in May 2004 in a Belgian hospital. Sequences identified a novel ESBL distantly related to other Ambler class A ESBLs. The *bla*_BEL-1_ gene was found as a gene cassette located in a chromosome-borne class 1 integron structure, named In120, bracketed by Tn*1404*-type transposon sequences and by *P. aeruginosa*-specific genes, containing three other gene cassettes (*aacA4*, *aadA5*, and *smr2*) (Poirel et al. 2005). Two other BEL variants have been described in Spain: BEL-2 on the chromosome of *P. aeruginosa*, with enhanced hydrolytic properties against expanded-spectrum cephalosporins, and BEL-3, with a single amino acid substitution (P160S) with respect to BEL-1 (Poirel et al. 2010)(Juan et al. 2010). This ESBL has been initially identified in *P. aeruginosa*, but subsequently on a non-conjugative plasmid in a *Klebsiella pneumoniae* isolate recovered from a patient in a Portuguese intensive care unit in 2009 (Papagiannitsis et al. 2015). BEL-1 has the characteristic of increasing the MIC of cefiderocol and ceftolozane/tazobactam when has been cloned into plasmid pUCp24 (Poirel et al. 2022). Cefiderocol is a novel siderophore cephalosporin with broad-spectrum activity including metallo-betalactamases (Falcone and Tiseo 2022), for which there are currently few therapeutic options (Doi 2019).

Horizontal transmission of this determinant has not been described to date and the appearance of mobile elements that facilitate its propagation among carbapenemase producers is a worrying fact. Additionally, the possibility of plasmid BEL enzymes conferring cefiderocol resistance is unknown. Recently, BEL and KPC co-producing *E. coli* isolates were detected over a 1-year period in the wastewater of a Spanish Hospital. The aim of this work was to characterize the genetic environments and plasmid carriage of these genes.

## Materials and methods

### Isolates

In a monitoring of wastewater from several hospitals in southern Spain (Romero-Oraá et al 2019), performed monthly, a total of 254 carbapenemase-producing Enterobacterales were identified over a 12-month period of study in Hospital Puerta del Mar in Cádiz, with 78 (31%) producing KPC-2. Of these, 19 (24%) were *E. coli*. Seven of these 19 isolates were co-producers of BEL and KPC enzymes and were detected, over the study period, in the months April, May, June, August, and October 2018 and January 2019. The hospital has 667 beds, carries out 150,000 stays/year and has 3 intensive care units, which are a reference for hospitals in the province. Samples (1 l) were collected between 10 and 11 h at the outfall of the hospital collector into the municipal sewer. Briefly, the isolates were recovered as follows: 1 ml of wastewaters, after a 10-min agitation to homogenize, were overnight enriched in peptone broth and then were platted on ChromID CARBA agar. Each different colony was identified by using matrix-assisted laser desorption ionization-time of flight (MALDI-TOF). Antimicrobial susceptibility testing was carried out by disc (Oxoid) diffusion agar method by using EUCAST breakpoints (https://www.eucast.org/clinical_breakpoints, 29 June 2023). Additionally, cefiderocol was also tested by using gradient strip (LiofilChem). Carbapenem resistance screening was performed with carbapenemics discs according to EUCAST guidelines and beta-carba test (Biorad). Carbapenemases were studied with NG Carba 5 lateral flow immunochromatography (NG Biotech), carbapenemases inhibitor discs and by PCR (GeneXpert) and ESBLs by double disc of cephalosporins with and without clavulanic according to EUCAST guidelines.

### Plasmid analysis

Plasmid analysis was carried out by two strategies. First, transconjugants containing of the BEL-1 plasmid from the 4 first isolates belonging to ST11685 clone were selected with *Escherichia coli* J53 in conjugation experiments by using MacConkey 4 mg/l cefotaxime as selective medium. All transconjugants were checked by PCR for *bla*_BEL-1._ Plasmid DNA was isolated by the method described by Kieser to check the number of plasmids in each transconjugant. Transconjugants were sequenced by Illumina at a × 300 coverage. Reads were mapped against *E. coli* J53 and unmapped reads were considered plasmidic and were assembled. Secondly, isolates 56 and 410, belonging each to one of the two clones identified, were sequenced using MinION MK1b device. Plasmid-based replicon typing was carried out by using PlasmidFinder and plasmid MLST of IncN plasmid was assigned by using pMLST 2.0. Relaxase typing was carried out by using MOB-typer (Robertson and Nash 2018). Plasmid sequences from Illumina were compared by using BLASTN with those previously reported in GeneBank (NCBI) and was plotted with the Easyfig program.

### Whole genome sequencing

Each *E. coli* isolate as well as each BEL-1 transconjugants were sequenced using an Illumina MiSeq platform and Nextera Flex DNA sample preparation kit. De novo assemblies were obtained using CLC genomics Workbench software (version 9.5.2). Minimum thresholds for contig size and coverage for *E. coli* isolates were set at 300 bp and 30 × , respectively, and for transconjugants coverage was set at 300 × . Assemblies were annotated using the tools of the Center for Genomic Epidemiology (https://cge.food.dtu.dk/services/): plasmid incompatibility group with PlasmidFinder 2.1 and antibiotic resistance determinants with ResFinder 4.1 with a threshold of 90% identity. Other annotations were carried out with Rapid Annotation using Subsystem Technology (RAST) server (http://rast.nmpdr.org/) and to determine insertion sequences was used ISfinder v2.0 (https://www-is.biotoul.fr). For identification of plasmid prophage sequence in p410N-BEL-1, the PHAge Search Tool Enhanced Release (PHASTER) (Arndt et al. 2016; Zhou et al. 2011) server was employed.

Moreover, isolates 56 and 410, belonging each to one of the two clones identified, were sequenced using MinION MK1b device (Oxford Nanopore Technologies, Oxford, UK). Libraries were prepared using the rapid barcoding kit (SQK-RBK004) and were loaded onto a R9.4 flow cell (Oxford Nanopore Technologies). Collection of raw electronic signal data and live base-calling was performed using the MinKNOW v1.4.2 (filtering criteria: length, > 1000 bp; quality > 8). Unicycler v4.8 was used to generated an hybrid genome with the MinION long-reads and short-reads from Illumina (Wick et al. 2017). Sequence homology searches (percentage BLASTN identity > 95%) were performed to identify similar plasmids. BLAST Ring Image Generator (BRIG) for plasmid comparisons (Alikhan et al. 2011). The complete nucleotide sequence of all isolates and plasmids were deposited publicly in NCBI under BioProject no. PRJNA1035043 (https://dataview.ncbi.nlm.nih.gov/object/PRJNA1035043?reviewer=jnoge5hv437qmjq7a42svid4tm).

### Typing

In silico analysis of MLST of the 7 isolates was implemented by MLST 2.0 available on the CGE website (identity = 100% and coverage = 100%). The Enterobase *E. coli* cgMLST scheme was employed to acquire the cgMLST type based on 2513 target *loci* of *E. coli* genomic sequences (Zhou et al. 2020) and Clermont’s phylogroup was assigned with phylotype experiment from Enterobase. SNPs calling among the 6 isolates belonging to ST11685 was done using CSI Phylogeny (Kaas et al. 2014) by using as reference strain the first isolate (isolate 56).

## Results

All 7 isolates were positive for *bla*_KPC-2_ and *bla*_BEL-1_ genes. All of them showed resistance to penicillins combined with beta-lactamase inhibitors, third-generation cephalosporins, ertapenem and nalidixic acid, being susceptible to fosfomycin, tigecycline, trimethoprim/sulfamethoxazole, amikacin, gentamycin, nitrofurantoine. All yielded positive imipenem hydrolysis and KPC positive reaction with NG Carba 5. All isolates belonged to phylogroup A and two different STs were observed: 6 isolates belonged to ST11685 (recovered from April to August 2018 and January 2019) and the other isolate belonged to ST2795 clones (recovered in January 2019). One of the ST11685 was susceptible to tobramycin, and the rest of isolates were resistant. The 6 isolates belonging to the same ST (ST11685) differed in 37–226 SNPs between them (Figure S1b). Three J53 transconjugants were obtained from clone ST11685 and one from the isolate of clone ST2795. All isolates and transconjugants were susceptible to cefiderocol (MIC range ≤ 0.03–0.25 mg/l). The transconjugants from ST11685 showed 1 dilution higher MIC value (0.125 mg/l) than J53 strain (0.06 mg/l) and the transconjugant from ST2795 yielded lower MIC value (≤ 0.03 mg/). All isolates and the three transconjugants were resistant to ceftolozane/tazobactam (MIC values > 2 mg/l).

Analyzing the hybrid genome obtained from both short-read and long-read sequencing methods, it was found that ST11685-56 harbored a circular sequence containing IncN-*bla*_BEL-1_ of 77,5 kb (p56N-BEL-1) belonging to ST24/MOB_F_ and a circular sequence containing IncP-*bla*_KPC-2_ of 43 kb (p56P-KPC-2). The comparison by using BLASTN of IncN-*bla*_BEL-1_ and IncP-*bla*_KPC-2_ from ST11685-56 with plasmid Illumina sequences from the other ST11685 isolates yielded a homology > 99% in all cases. Additionally, in one of the ST11685 isolates was also detected an *Inc*X5 replicon (Figure S1a). The ST2795-410 hybrid genome showed a larger IncN-*bla*_BEL-1_ of 109 kb (p410N-BEL-1), also belonging to ST24/MOB_F_ and a IncP-*bla*_KPC-2_ (p410P-KPC-2) of similar size (39 kb) than ST11685 isolates. Additionally, ST2795-410 strain harbored two other plasmids which did not contain any *bla* gene: a IncF plasmid of 71 kb with formula F100:A-:B- and another plasmid of 251 kb which showed > 99% homology with 173 kb of a plasmid of pKPC-CAV1321 found in a *C. freundii* isolate detected in UK (Accession no. CP011611) (14).

**Fig. 1:**
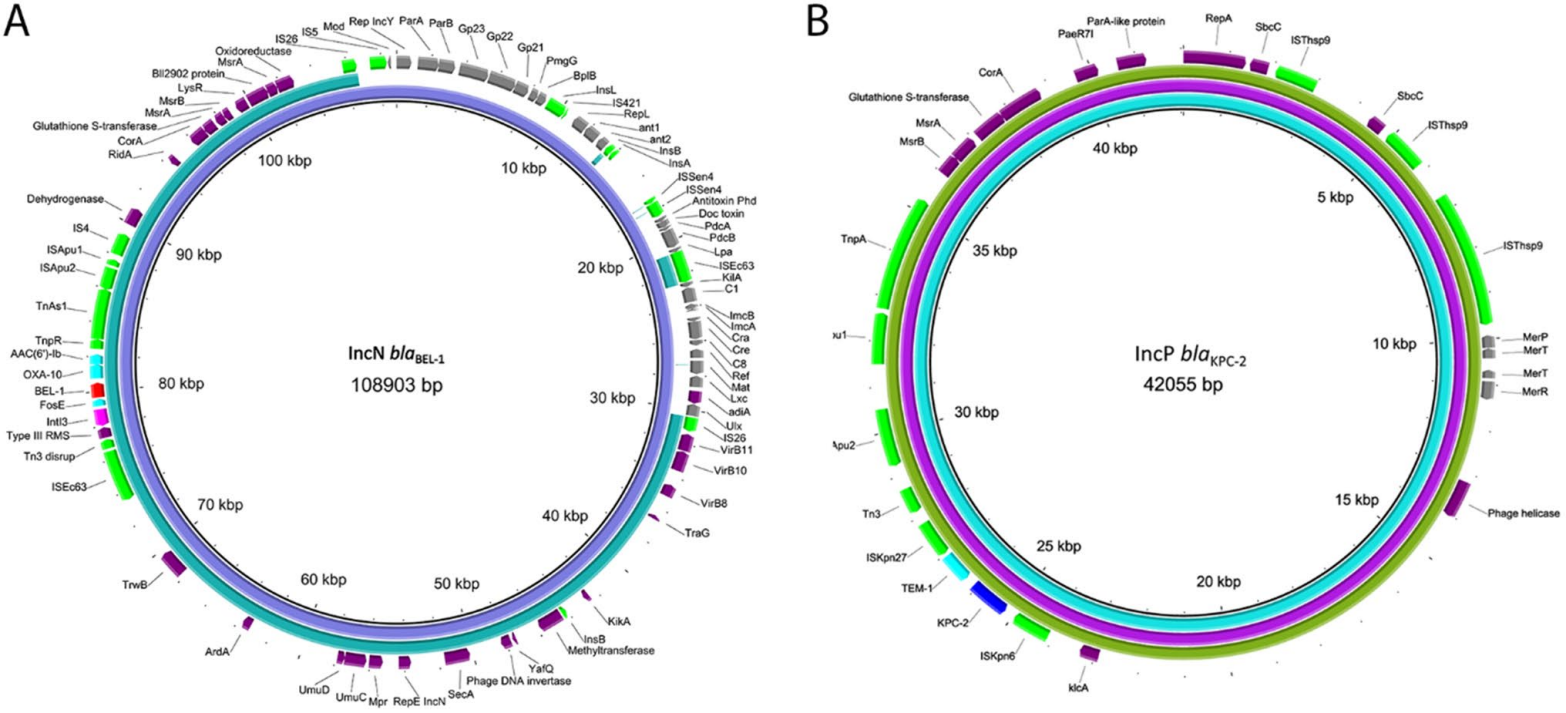
**A** BRIG comparison of the two IncN plasmids harbouring *bla*_BEL-1_ from *E. coli* strains analyzed in this study. Each plasmid is a ring and the larger p410N-BEL-1 was used as reference. The layout of *bla*_BEL-1_ is highlighted with a red arrow. Other antibiotic resistance determinant arrows are highlighted in blue, the insertion sequences are highlighted in green, P1-like phage sequences in gray and other genes are in purple. 1B) BRIG comparison of pA1705-KPC (blue ring) with the two IncP plasmids harboring *bla*_KPC-2_ from *E. coli* strains analyzed in this study, and pA1705-KPC (blue ring) was used as reference. The layout of *bla*_KPC-2_ highlighted with a blue arrow, the operon *mer* genes in gray, and the rest of genes in the same colors as in **A**

Comparison of the two IncN-*bla*_BEL-1_ plasmids from each *E. coli* strain showed a shared region of 76,2 kb (99% identity) which contained the *bla*_BEL-1_ environment (Fig. 1A). According to the Unicycler results, the coverage of the p56N-BEL-1 and p410N-BEL-1 plasmids, standardized to the chromosome contig, was 2.31X and 3.61X, respectively. Additionally, the p410N-BEL-1 incorporated phage sequences (23 kb in total) which were shown a 98% of homology to P1-like bacteriophage RCS47 (accession no. NC_042128) (15). The existence of the N plasmid sequences alongside the P1-like sequences was verified by analyzing only reads > 20,000 bp (Figure S2). The p410N-BEL-1 included the characteristic *repA* of IncY of this phage as well as some genes of the baseplate and tail, including the lytic RepL and lysogenic proteins, but some parts of the 5 characteristic regions of this type of phages are missing. In the P-1-like part of the plasmid the region 1 had the *mod* gene disrupted by *IS*5, important region 3 structures were absent (c-segment, baseplate and tube genes, and immunity-associated genes) as well as region 5 genes related to head structure and processing. IncN-*bla*_BEL-1_ plasmids exhibited a completely different backbone than the previously described 12 kb- pKP-M1144 found in *K. pneumoniae* in Portugal. A comparison of both IncN plasmids with those deposited at NCBI revealed a 99.7% homology with a 48.3 kb region in a IncN plasmid (accession no. NZ_LT599827) carrying *bla*_KPC-2_ gene, found in an *E. coli* ST362 isolate detected in Germany in 2010. On the other hand, the comparison of IncP-*bla*_KPC-2_ plasmids from the two *E. coli* strains yielded 99,9% of identity between them. These plasmids were identical to pA1705-KPC (accession no. NZ_MH909348) by BLASTN, a plasmid of 42 kb detected in a *K. pneumoniae* in China (Fig. 1B). In addition to the *bla*_KPC-2_ gene flanked by *IS*Kpn6 and *IS*Kpn7, all these IncP plasmids, including the one from China, carried the *bla*_TEM-1_ and *mer* operon genes.

**Fig. 2:**
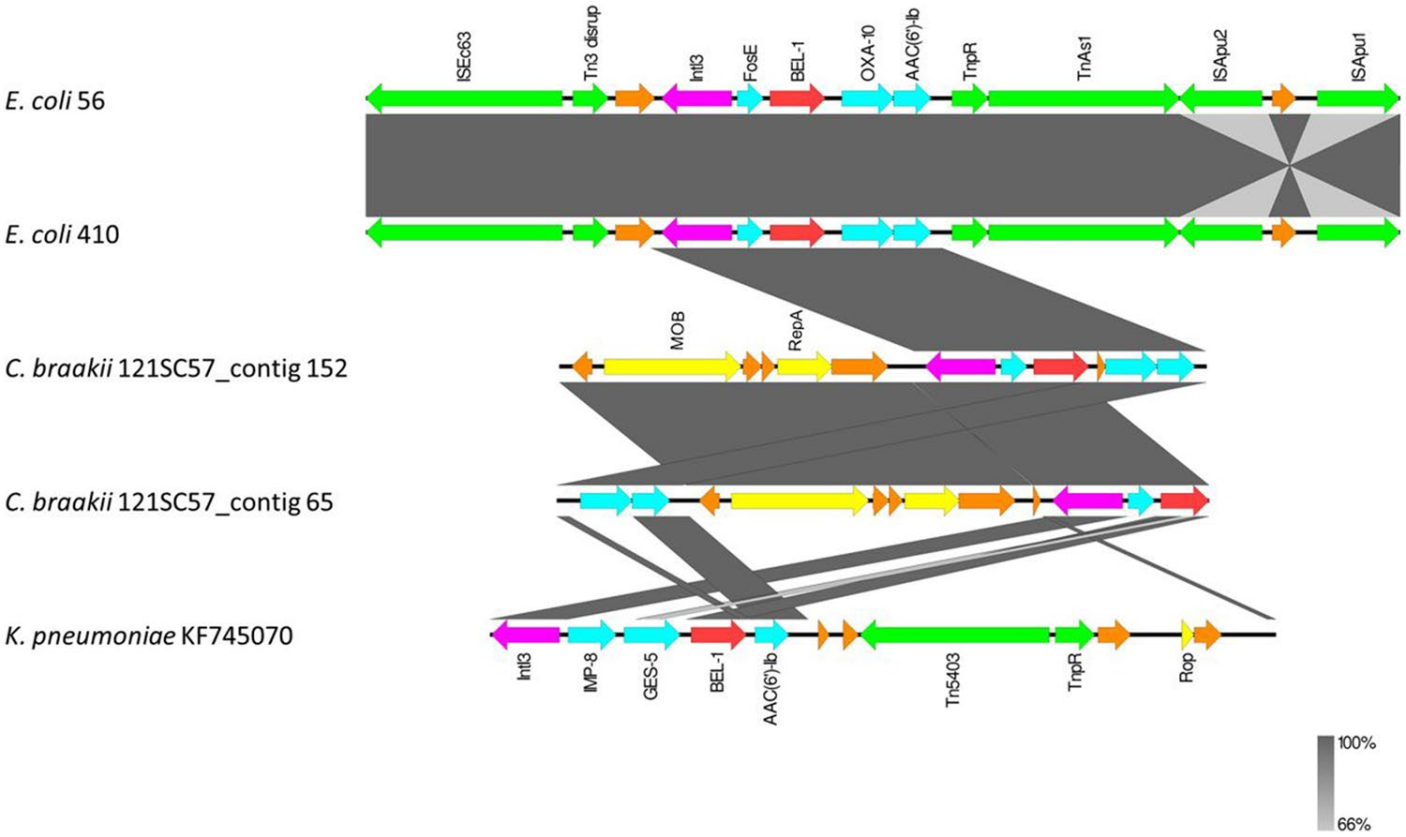
Comparison of *bla*_BEL-1_ containing genetic environments of Enterobacterales compared in this study. Protein coding regions are represented by arrows indicating the direction of transcription and colored as follows: BEL-1 in red, other resistance determinants in blue, integrase in pink, insertion sequences and transposases in green, the relasaxe and RepA protein in yellow, and hypothetical proteins in orange. The gray shedding reflects nucleotide sequence identities. The alignments were represented with EasyFig v2.2.5

Both in ST11685-56 and ST2795-410 *E. coli* strains, the *bla*_BEL-1_ gene was found embedded in a class 3 integron. Surrounding the *bla*_BEL-1_ gene, the gene *fosE* was found upstream and *bla*_OXA-10_ and *aac(6’)*-1b genes were identified downstream. In addition, this class 3 integron was surrounded by Tn*3* transposon sequences. This cassette was found to be homologous by BLASTN search to another structure found in a *Citrobacter freundii* complex isolate (121SC57), collected from a sewage sample in Spain in 2012 (Accession no. DACUGO010000000.1) (16). Within this isolate, two copies of the class 3 integron structure containing the *bla*_BEL-1_ gene, with the same gene content that in our *E. coli* strains, were found (Fig. 2). The chromosomal or plasmid location of these two copies in the *C. freundii* isolate is unknown as the contigs where they were found were too small, but the *mobA* and *repA* genes were also present in the contigs of both copies and could indicate a possible plasmid localization. The analysis of the *mobA* sequence assigned these two contigs to MOB_Q_ protein family. Additionally, analyzing the plasmid content of the *C. freundii* genome using PlasmidFinder the following Col440I, IncFIB(K), IncN, IncP6, IncR and pKPC-CAV1321 replicons were identified.

When the Portuguese *K. pneumoniae* isolate was compared with the *E. coli* BEL-1 platforms, the *bla*_BEL-1_ gene was similarly located within an identical class 3 integron*,* but in this case in a 12 kb ColE-1 like plasmid (pKP-M1144, accession no. KF745070), and the integron contained a *bla*_IMP-8_ and a *bla*_GES-5_ genes upstream the *bla*_BEL-1_ gene, *fosE* and *bla*_OXA-10_ were not found and a Tn*5403* was detected downstream (Fig. 2). Finally, also using BLASTN search, an isolate of *Raoultella ornithinolytica* (accession no. DACSDR010000000.1) carrying BEL-1 gene was found, having this genome a short contig containing only the BEL-1 gene and a IS*6100* insertion sequence.

## Discussion

This is the first description of BEL-1 and KPC-2 co-produced in Enterobacterales and the first detection of this enzyme in *E. coli*. Although the origin of this combination is unknown, it is most probable that these strains originated from the discharge of a patient. However, both the clonal relationship of the isolates and the characteristics of the plasmids and cassettes may also indicate the possibility that that they may be the result of gene transfers and rearrangements in a human spill environment, rather than directly from patient discharges. IncN-BEL-1 plasmids showed some environmental characteristics. Firstly, the incompatibility group IncN is very abundant in wastewater of human origin, both municipal and hospital sources. In a study conducted in the La Paz River basin in Bolivia, where hospitals, industries, and households discharge directly, all the plasmids detected from the urban site were IncN (Guzman-Otazo et al. 2022). A comparative study of the inlet of a municipal wastewater treatment plant (WWTP) and the main sewer line of a Swedish hospital also revealed that IncN plasmids constituted the majority of the microbial population (Hutinel et al. 2021). This group of plasmids are self-conjugative, common in Enterobacterales and they had been harbored ESBLs, carbapenemases, and aminoglycoside-modifying enzymes (García-Fernández et al. 2011). On the other hand, IncP-KPC-2 plasmids are broad-spectrum conjugative plasmids, and have been localized in Enterobacteriaceae recovered from hospital waste in Japan (Ota et al. 2022).

The second notable feature is the type 3 integrase carrying *bla*_BEL-1_ that we found in our isolates, which is also present in the previous *C. freundii* and *K. pneumoniae* isolates. To date, the *bla*_BEL-1_ has usually been found in cassettes with class 1 integrons in *P. aeruginosa* (Bouheraoua et al. 2018)*,* and had only been observed associated with the class 3 integrase in the previously reported *K. pneumoniae* isolate from Portugal (Papagiannitsis et al. 2015). The class 3 integrase is not frequent in collections of multidrug-resistant isolates and has not been detected in isolates from healthy volunteers in Spain (Vinué et al. 2008) or in a population-based study in France (Laroche et al. 2009). In contrast, in a study of wastewater samples from a municipal treatment plant in Canada (Jankowski et al. 2022), the class 3 integrase was found almost as frequently as class 1, which reinforces the hypothesis that the cassette with  BEL-1 is found in isolates adapted to wastewater. When we compared the genetic environment of *bla*_BEL-1_ from our environmental isolates and the one from the clinical Portuguese isolate, the differences found in the two class 3 integrase cassettes may indicate different captures or two different evolutions of this cassette.

Finally, the *E. coli* strains are detected in discharges of a hospital where no BEL-1 or KPC-2-producing isolates were detected before or during that period neither have been submitted to the regional reference laboratory of Andalucía from other hospitals, where submissions are voluntary. It cannot be ruled out that there are colonized or infected patients and that they have not been detected, since not all *E. coli* isolates recovered from the hospital have been characterized at the molecular level. The persistence of one of the *E. coli* strains throughout the year, albeit with small differences between isolates, also supports the possibility that these isolates could be part of the collector biofilms.

Parallel to the arguments supporting a human sewage origin, a feature that may indicate a human origin of *bla*_BEL-1_ gene is the presence of P1-like phage sequences in the plasmid of one of the two strains. P1-like prophages can behave as a plasmid due to a plasmid replicon which belongs to plasmid incompatibility group (Inc) Y, and several reports have associated P1-like elements with *mcr-1* and ESBL genes (Billard-Pomares et al. 2014). What has been described to date are isolates that carry P1-like phage-plasmids in addition to other plasmids. Fusions of plasmids and P1-like phage-plasmids have only previously been demonstrated in an *E. coli* isolate obtained from a fecal sample of an inpatient in China (Bai et al. 2017). On this occasion, it was found the fusion of IncH12 with P1 and, similar to our strain, the phage sequences were incomplete.

Finally, it remains to comment that these isolates are susceptible to cefiderocol despite the production of BEL-1. The presence of this enzyme had been associated with a 1 dilution-increase in the MIC value of cefiderocol when this gene was cloned into pUCp24 (Poirel et al. 2022). A similar increase was observed in some of the transconjugants of clone ST11685, but this increase does not seem to confer resistance.

## Conclusions

In conclusion, we have found the presence of BEL-1 producing *E. coli* isolates associated with KPC-2 production in hospital discharges with some genetic characteristics typical of human wastewater resident flora and in a different cassette than usually observed in *P. aeruginosa*. Broader epidemiological studies, including environmental samples from other non-human sources, would be necessary to determine the origin of this new BEL-1 cassette.

### Supplementary Information

Below is the link to the electronic supplementary material.

Supplementary file1 (XLSX 11 KB)

## Data Availability

All datasheets of this study will be available at https://idus.us.es after publication.
